# Syphilis ascendant: a brief history and modern trends

**DOI:** 10.1186/s40794-016-0039-4

**Published:** 2016-09-26

**Authors:** Wesley G. Willeford, Laura H. Bachmann

**Affiliations:** 1 Department of Internal Medicine, Wake Forest Baptist Health, Section on General Internal Medicine, 1 Medical Center Blvd, Winston-Salem, NC 27101 USA; 2 Department of Internal Medicine, Wake Forest Baptist Health, Section on Infectious Diseases, 1 Medical Center Blvd, Winston-Salem, NC 27101 USA

**Keywords:** Syphilis, Men who have sex with men, Congenital syphilis

## Abstract

To provide a miniature review of recent literature surrounding a brief history of syphilis, to discuss the recently increasing incidence of syphilis, to discuss recent United State Preventative Service Task Force recommendations for syphilis screening, and to discuss congenital syphilis.

The literature review was conducting using PubMed with the following search terms: syphilis, congenital syphilis, MSM and syphilis, prenatal syphilis, neurosyphilis, and other related terms.

*Treponema pallidum* has been a constant, and unwanted, companion of humankind since antiquity. This sexually transmitted infection (STI) has the potential to affect virtually every rung of society—young and old, rich and poor, but it has a proclivity for the most vulnerable groups among us. Since record high rates of infection in the World War II era, tremendous progress has been made in effectively controlling the infection, and this has been largely mediated by the efficacy of penicillin on the causative spirochete. However, 2014 data from the Centers for Disease Control and Prevention demonstrated a sharp increase in the rate of new cases of syphilis, predominantly in men who have sex with men. Additionally, the numbers of newly diagnosed cases of congenital syphilis are on the rise as well.

In effect, a burgeoning crisis has come to the doorstep of the medical community. We are faced with changing attitudes regarding sexual interactions. The authors believe that geolocation dating and sex applications for smart phones increase the availability of sexual encounters. Pre-exposure prophylaxis may be leading to more *laissez-faire* attitudes toward unprotected intercourse, and with increased opportunities for sexual encounters, co-infected states with other diseases may be altering the landscape of STIs.

In 2016, in response to increasing rates of newly diagnosed syphilis, the United States Preventative Health Services Task Force reaffirmed the need for syphilis screening in at-risk populations. However, primary care physicians and advanced practice providers may not always be aware of which patients fall into that category. Due to the highly personal nature of discussing sexuality, sexual behavior may not be explored at all.

Numerous challenges lie ahead of the infectious diseases, primary care, and public health communities in attempting to bend the curve of the ascendant rise in syphilis. To adequately combat this infection, sufficient funding will need to be provided to public health departments, adequate access to health care resources will be needed to allow for the necessary screening of patients, and primary care practitioners will need thoroughly engage with their patients to understand their sexual practices and to offer the necessary interventions.

*Treponema pallidum* has affected the lives of millions in all social strata. It has been speculated that various artists, writers, and great historical figures, ranging from Oscar Wilde and John Keats to Henry VIII and Adolf Hitler, were touched by the disease [1]. Whether a person is of high or low socioeconomic class, syphilis does not discriminate. However, the groups most widely affected vary by the era and the social circumstances of the time [2]. The demographics of the disease have changed substantially over the course of the last century, and even today, syphilis has seemingly come back from the brink of defeat; this old nemesis of humanity has come out swinging.

The word “syphilis” was coined by Giraloma Fracastoro in 1530 in his book *Syphilis sive morbus gallicus*. Once a name had been assigned to the general syndrome of the illness, various attempts at discovering a cure were made throughout the proceeding centuries, though a lasting treatment was not discovered until 1943 with the arrival of a nearly true “silver bullet,” penicillin. A rarity in the interplay of antibiotics and bacteria, penicillin has remained effective in the treatment of *T. pallidum* since its introduction, and it continues to be an efficacious first line therapy [3]. Withthis therapy in hand, the defeat of syphilis as a sexually transmitted infection (STI) seemed like a feasible task to such an extent that the Centers for Disease Control and Prevention (CDC) enacted the Syphilis Elimination Effort; however, this was suspended in December 2013 due to a sharp upturn in the incidence of the disease. While the current incidence of syphilis infection is not as high as seen in the mid-to-late 1940s, the incidence has been on the rise since the early-to-mid 2000s. As previously alluded, the face of who acquires syphilis has been in flux. After World War II, the spread of the disease was often mediated by GIs returning from the warfront, and in the late 1980s and early 1990s, the groups most affected by syphilis changed from a heterosexual epidemic to one in which men who have sex with men (MSM) began to comprise an increasingly larger proportion of newly diagnosed cases [2].

According to the CDC, in 2000 and 2001, the national rate of reported primary and secondary syphilis cases in the United States was 2.1 cases per 100,000 population (6103 cases reported); this represented the lowest rates since 1941. As of 2014, the incidence increased to 6.3 cases per 100,000 population (19,999 cases reported) [4]. The majority of these new cases were in MSM populations, and multiple smaller studies have reported similar results [5, 6]. Patton et al. reported a startling rate of 50–70 % HIV co-infection among MSM infected with primary and secondary syphilis [6]. Another group adversely affected by overall increases in the rate of syphilis is newborns. According to Bowen et al., congenital syphilis in the United States has increased from a rate of 8.4 cases per 100,000 live births (334 cases) between 2008 and 2012 to a rate of 11.6 cases per 100,000 live births (448 cases) between 2012 and 2014 [7]. Bowen et al. go on to postulate that these increases may be due to lack of widely available and easily accessible prenatal care for at-risk populations as their data revealed that 100 out of 458 mothers of infants with congenital syphilis received no prenatal care; furthermore, of those who did receive care, 62 mothers had tested positive for syphilis and did not receive any treatment [7]. At present, the CDC recommends screening for syphilis upon entry to prenatal care, and then again during the third trimester if the patient is at high risk [3].

What societal factors are driving the recent epidemiological changes, especially in MSM populations? This could partially be partially mediated by changing perceptions of HIV infection in general, its lethality, and the introduction of pre-exposure prophylaxis (PrEP). Kojima et al. have found that MSM populations taking PrEP for HIV prevention are up to 44.6 times more likely to contract syphilis infection than MSM populations who are not on PrEP [8]. Additionally, we believe that fundamental shifts are occurring in the availability of opportunities for sexual activity in the form of dating and sex location applications for smart phones. Beymer et al. report greater odds of contracting gonorrhea and chlamydia among MSM clients who used networking applications, and on a larger scale this could, in theory, be extrapolated to other STIs, due to the easy availability of sexual encounters [9].

Beyond changes in the incidence of syphilis, presentations of the disease may be changing as well. Between December 2014 and March 2015, the CDC reported four cases of ocular syphilis from King County, Washington, and eight cases from San Francisco, California [10]. A manifestation of neurosyphilis, this process can result in loss of sight due to anterior, posterior, or panuveitis; prompt diagnosis and treatment can mitigate the degree of vision loss [11]. However, the medical community is left grappling with whether this is a manifestation of increasing incidence of syphilis or the effect of concomitant immunodeficiency. The answer to this question is not immediately clear; time and further research will be required to arrive at a conclusion. Ocular syphilis has also disproportionately affected MSM, and this further reinforces the previously demonstrated need for adequate sexual history taking and screening in all populations, but particularly for men [12, 13]. Metcalfe et al. report unsettling survey data which demonstrates up to 60 % patients who were MSM had not revealed their sexual practices to their general practitioners (GP), and a significant proportion this group reported that their GP had never asked them about their sexual activities [14].

As the incidence of syphilis has increased in such a robust fashion, new recommendations from the United States Preventive Services Task Force (USPSTF) were released in 2016. These recommendations reaffirmed the need for syphilis screening in high-risk populations (MSM and men and women with HIV) [15]. As clinical experience with syphilis has decreased with the overall decreasing incidence since the 1940s post-World War II high, clinicians are not as well-versed in the subtle and myriad findings of syphilis. In oft-quoted maxim of medicine, Sir William Osler said, “He who knows syphilis, knows medicine,” and as a disease that can manifest in numerous locations throughout the body, vigilance and suspicion must remain high. Taking a careful sexual history is the first step to developing a comprehensive evaluation and management plan. The reality is that, as alluded to above, the sexual history is often neglected due to discomfort with the subject material, and providers may stray away from discussing such issues [16]. However, as an integral component of a holistic approach to sexual health, it is important that the sexual history become a routine part of clinical care, thereby enhancing both patient and provider comfort with the subject matter. Because syphilis often has subtle findings on presentation, physicians should also perform a detailed physical examination including evaluation of all anatomical sites, regardless of reported sexual activity, as well as a neurologic exam.

In conclusion, syphilis is on the rise, and a multi-pronged strategy is required to bend the curve of this public health crisis, which is disproportionately affecting MSM populations and infants—already vulnerable groups. Integral to that strategy is the USPSTF recommendations for screening for syphilis in high-risk populations to better estimate the actual burden and extent of the current crisis and to allow for timely treatment to prevent further spread. Beyond that, as clinicians, we have to continue to engage with our patients to understand their sexual behaviors so that we can offer counsel, services, and the necessary testing to provide for their wellness. Additionally, clinicians should continue to strive to empower their patients to be advocates for their own health, to know their STI status, and to understand what can be done to minimize their risks. Perhaps the greater challenges that must be better understood are the impact of mobile phone applications on the spread of STIs, the perceptions of MSM on PrEP regarding safe sex and health screening, and the interplay of HIV and syphilis in a co-infected state. If PrEP is truly driving a change in views and attitudes toward STIs, as a community of scientists and clinicians, we need to continue to describe these attitudes and understand patient motivations to begin to effect meaningful change. In addition, it is likely that different strategies will be required to address the increased rates of syphilis in women, which is directly tied to the recent increases in congenital syphilis. Access to reliable and high-quality prenatal care is paramount along with partner testing—especially when we consider how grave an impact congenital syphilis has on the course of an individual’s life. Attention should be given to proper care with syphilis screening occurring at entry into prenatal care and, in high risk mothers and/or high prevalence settings, at the 28^th^ week of gestation and at the time of delivery [3, 17]. The time for action is at hand, and with luck, we will see a return of historically low incidences of syphilis throughout the world.
